# Color polymorphism in a land snail *Cepaea nemoralis* (Pulmonata: Helicidae) as viewed by potential avian predators

**DOI:** 10.1007/s00114-013-1049-y

**Published:** 2013-05-08

**Authors:** Adrian Surmacki, Agata Ożarowska-Nowicka, Zuzanna M. Rosin

**Affiliations:** 1 Department of Avian Biology and Ecology, Faculty of Biology, Adam Mickiewicz University, Umultowska 89, 61-614 Poznań, Poland; 2 Adam Mickiewicz University Botanical Garden, Adam Mickiewicz University, Dąbrowskiego 165, 60-594 Poznań, Poland; 3 Department of Cell Biology, Faculty of Biology, Adam Mickiewicz University, Umultowska 89, 61-614 Poznań, Poland

**Keywords:** Crypticity, Background matching, Camouflage, Color vision, Frequency-dependent selection

## Abstract

Avian predation is one of the most probable factors maintaining polymorphism of shell coloration in *Cepaea nemoralis*. This assumption is justified by the fact that birds frequently forage on snails and their prey choice varies with morph coloration. However, in all preceding studies, the conspicuousness of morphs was determined only by using human vision which is significantly different from birds’ visual perception. In this study, we assessed how birds perceive colors of four *Cepaea nemoralis* morphs using physiological models of avian color vision. We calculated combined chromatic and achromatic contrast between shells and three habitat background types as a measure of shell conspicuousness. The degree of background color matching in *Cepaea nemoralis* depended on both shell morph and habitat type. On average, banded morphs were more conspicuous than unbanded morphs. Morphs were the most cryptic against dry vegetation and the most conspicuous on bare ground. We also found a significant interaction between habitat type and color morph. The relative conspicuousness of shell morphs depended on habitat and was the most variable against green vegetation. Our study provides the first insight into how potential avian predators view *Cepaea nemoralis* morphs. The results are discussed in light of multiple hypotheses explaining selective predation on *Cepaea nemoralis* morphs.

## Introduction

Predation is one of the most intensively studied agents responsible for the maintenance of color polymorphism in animals (Endler 1986; Ruxton et al. 2004; Bond 2007). Indeed, polymorphic species are abundant in many prey organisms like snails (Byers 1990), spiders (Théry and Casas 2002), moths (Barnes and McDunnough 1918), crabs (Krause-Nehring et al. 2010), or locusts (Pellissier et al. 2011). Generally, it is assumed that predation can maintain color polymorphism in two ways: selection for crypsis and apostatic selection (Clarke 1969; Endler 1978; Bond 2007). The heterogeneity of the environment where the prey species evolved is thought to influence the evolution of crypsis and apostatic selection. Heterogeneous areas that consist of large patches of diverse habitats (“coarse grain habitats”; Levins 1968) will promote the evolution of specialist morphs through selection for crypsis (Endler 1978; Bond 2007). The output of the process is a few distinct morphs, each well matched to the coloration of the preferred habitat type (“specialist polymorphism”; Bond 2007). In homogenous areas that are a mixture of small microhabitats (“fine grain habitats”; Levins 1968), apostatic selection is more likely to evolve. Species living in such circumstances tend to evolve multiple distinctive morphs. Because they frequently move across all microhabitats, they evolve coloration that is equally cryptic in all “grains” of the habitat (“generalist polymorphism”; Bond 2007). In such circumstances, predators use search images of the most common morph, and this can lead to frequency-dependent selection (Clarke 1962; Bond 2007).

In studies testing selection for crypsis, one of two (or both) of the following approaches is commonly used: (1) comparison of survival of prey morphs (often artificial or virtual) exposed to real predators against different backgrounds and (2) examining how well morphs match the background of their habitat. In studies focusing on apostatic selection, the frequency and the characterization of morphs in natural (Fitzpatrick et al. 2009), laboratory (Cook and Kenyon 1991), or virtual conditions (Bond and Kamil 2002) are manipulated, and then, survival is scored. It is worthwhile to point out, however, that selection for crypsis and apostatic selection are not mutually exclusive. Both kinds of selection (i.e., apostatic selection and selection for crypsis) are possible at the same time when exerted by two different kinds of predators (see McKillup and McKillup 2008 for a classic example). Another possibility is that prey species occur in area encompassing both “fine” and “coarse grained” habitats. Under such circumstances, evolution of both “generalist” and “specialist” morphs is possible, respectively.

Yet, the possibility that both selection for crypsis and apostatic selection are acting in tandem is rarely considered within a single study (for exceptions, see Cook 1986; Cook and Kenyon 1991; Bond and Kamil 2006; McKillup and McKillup 2008). The apostatic selection theory states that morphs are equally cryptic against the same substrate (generalist polymorphism; Bond 2007). This assumption is either ignored or arbitrily assumed without testing, and studies have been designed in a way that totally excludes the potential for interactions between natural habitat and morph crypsis (e.g., Allen and Clarke 1968; Allen 1974; Cook and Miller 1977; Allen and Weale 2005).

One of the most intensive studies of predator-driven selection on polymorphic species focused on the brown-lipped grove snail *Cepaea nemoralis* (L.) and their avian predators (e.g., Cain and Sheppard 1950; 1954; Clarke 1969; Allen and Weale 2005; Punzalan et al. 2005; Rosin et al. 2011). This snail species exhibits genetic variability in shell color (yellow, pink, or brown) and banding pattern (zero, one, three, or five dark bands), creating a dozen or so morphs (Richards and Murray 1975). Many factors appear to influence spatiotemporal variability in *Cepaea* shell color including genetic drift, migration, climatic selection, habitat heterogeneity, and landscape structure (e.g., Jones 1974; Hutchison and Templeton 1999; Cameron and Pokryszko 2008; Le Mitouard et al. 2010; Ożgo 2012). Selective predation by birds has long been hypothesized as one of the main forces shaping polymorphism in shell coloration of *Cepaea nemorali*s (e.g., Cain and Sheppard 1954; Allen 2004; Cook 2005; Punzalan et al. 2005; Rosin et al. 2011), but the exact mechanism behind this process remains uncertain. Some bird species, mainly thrushes Turdidae, specialize in eating snails and have developed behavioral adaptations that enable efficient shell breaking (Morris 1954; Cameron 1969). To date, most studies have focused on the role of apostatic selection and have provided evidence both in support of and against this hypothesis (Clarke 1962; Allen and Clarke 1968; Clarke 1969; Allen and Weale 2005). On the other hand, the idea that variation in crypsis among *Cepaea* morphs influences the level of predation pressure has received markedly less attention (Cain and Sheppard 1950; 1954; Cain 1983; Cook 1986).

Considering the “fine-grained” nature of *Cepaea nemoralis* habitats and their multiple morphs, apostatic selection driven by birds seems to be the most probable mechanism of predation-driven selection. However, we cannot exclude the possibility that, within the same habitat, some morphs are more cryptic than others. Thus, avian predation pressure selects for crypsis and favors morphs that match the preferred microhabitat. This would be possible if the morphs’ microhabitats are spatially or temporary separated. For example, for most of the year, psammophilic habitats may be dominated by dry (yellow) vegetation, while meadows are likely dominated by live (green) vegetation. Moreover, within the same area, dry vegetation occurs in the early and late seasons, while live vegetation dominates during mid season.

Our understanding of how avian predation could affect *Cepaea nemoralis* populations requires knowledge of their relative cryptic properties in the context of natural habitats. Despite extensive studies on *Cepaea nemoralis*, morph coloration has never been quantitatively assessed, and all determinations of conspicuousness were made solely by the visual ability of humans. In this study, we measured background matching (*sensu* Merilaita and Stevens 2011) of four morphs of *Cepaea nemoralis* in three common microhabitats using physiological models of bird vision (Vorobyev and Osorio 1998; Vorobyev et al. 1998). The method is commonly used in studies of prey–predator relationships (e.g., Stuart-Fox et al. 2004; Darst et al. 2006; Farallo and Forstner 2012). It allows researchers to assess differences in color (chroma) and brightness of two objects (snail shell and microhabitat substrate) when viewed by particular observers (birds). According to apostatic selection theory, crypsis of all morphs should be similar within one microhabitat. If birds exert selection for crypsis on *Cepaea nemoralis*, then we should expect the specialization of morphs to match a particular microhabitat better than others.

## Materials and methods

### General

We used 105 individuals of brown-lipped banded snail belonging to four color morphs: pink unbanded (hereafter P0, *n* = 26), pink with five bands (hereafter P5, *n* = 21), yellow unbanded (hereafter Y0, *n* = 34), and yellow with five bands (hereafter Y5, *n* = 24). We excluded other morphs for two reasons. First, the brown morph was absent in the study area. Second, we attempted to choose the morphs with the most distinctive background color. Third, pink and yellow morphs with one and three bands have shell background color that is very similar to respective morphs without bands (Rosin, Z.M., unpublished data). Individuals of *Cepaea nemoralis* were collected in July 2009 from a population located near the city of Poznań, Wielkopolska, Poland (52°26′ N, 16°52′ E). The sampling site covered 300 m^2^, which is less than the estimated size of one panmictic unit in *Cepaea nemoralis* (400 m^2^; Lamotte 1951). The collection of snails was random across the sampling site. Approximately 24 h after collection, snails were euthanizeed by placing them into a freezer (−23 °C) for 4 weeks. Background samples were collected in July 2012 at a different site than snails (52°28′ N, 16°55′ E, 4.76 km apart). Habitats in both sites were very similar, consisting of open areas of grass, herbaceous vegetation, patches of bare ground, and scattered low broadleaved trees and bushes including *Prunus spinosa*, *Robinia pseudoacacia*, and *Acer campestre*. Moreover, in both areas, *Cepaea nemoralis* is abundant (Rosin, Z.M., unpublished data). We collected samples of three types of the most common habitat backgrounds where snails were spotted: living (green) plants, dry plants, and bare ground. We collected three specimens of living plants belonging to 17 species common in the area: *Deschampsia caespitosa*, *Cirsium arvense*, *Cirsium oleraceum*, *Plantago major*, *Artemisia vulgaris*, *Soilidago canadensis*, *Taraxacum officinale*, *Vicia cracca*, *Potentilla anserina*, *Phleum pratense*, *Trifolium repens*, *Trifolium pratense*, *Festuca pratensis*, *Bromus inermis*, *Agropyron repens*, *Urtica dioica*, and *Festulolium adscendens*. Samples of dry (dead) plants (*n* = 20) were collected from stems and leaves of unidentified grasses, which were the dominant dead vegetation type in the area. Samples (2 × 2 cm^2^) of soil (*n* = 20) were cut from the ground surface. Sites from which samples of dry plants and soil were taken were selected randomly, but were no closer than 10 m apart. The total collection area was 2.2 ha.

### Spectrometry

We used a USB4000 spectrometer and a pulsed xenon lamp (PX2, Ocean Optics, Dunedin, FL, USA) connected with a fiber optic measuring probe (FCR-7UV200-2-1,5x100, Avantes, Eerbeek, The Netherlands). Using a 90° incident and measurement angle, we fixed the distance from the object surface at 1.5 mm. Before measuring each individual (or background sample), we standardized measurements using a white standard (WS-1-SL, Labsphere, North Sutton, NH, USA), while the dark standard was taken by turning off the light source and covering the probe. Spectral measurements of shells and habitat backgrounds were expressed as percent of light reflected at different wavelengths (Figs. 2 and 3). We processed spectral data using RCLR v0.9.28 software (Montgomerie 2008).

**Fig. 1:**
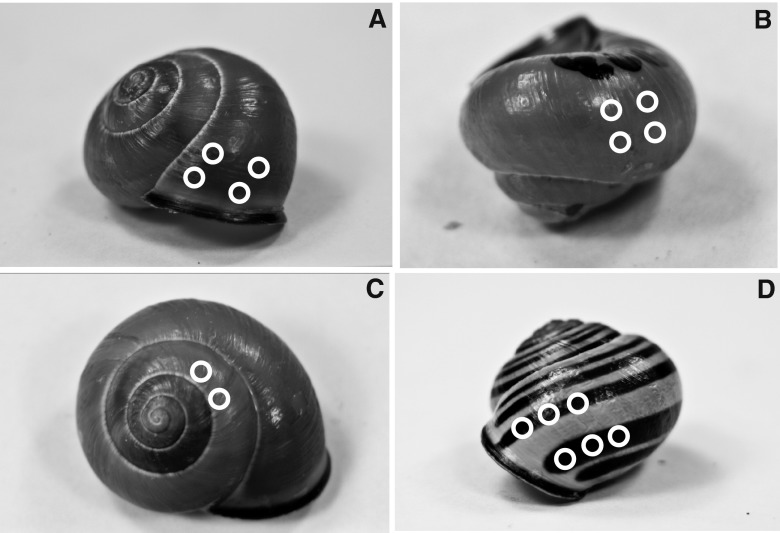
Reflectance measurement locations of shell background (**a**, **b**, and **c**) and bands (**d**). *Black patches* on picture **b** are snail ID numbers written onto the shell

Each plain morph (Y0 and P0) individual was measured in 10 locations (Fig. 1). The shells of the banded morphs (Y5 and P5) were measured in 16 locations: 10 locations on the shell background and 6 locations on bands. Locations of the shell background (*n* = 10) were measured in the same way as in unbanded morphs (Fig. 1). Typically, those were in the gaps between the second and third bands and between the third and fourth bands (Fig. 1a, b) and anterior to the first band (Fig. 1c). We measured the third and fourth bands, which were usually the thickest (Fig. 1d).

We took five reflectance measurements from each plant specimen, yielding a total of 15 measurements for each species. We took five reflectance measurements from each soil and dry plant sample. Green plants were placed on black velvet, while dry plants were layered prior to measurements. In all cases, the probe was moved at least 2 mm before each measurement. Reflectances taken from one shell and each of the habitat background types were averaged prior to further analysis.

### Visual models

**Fig. 2:**
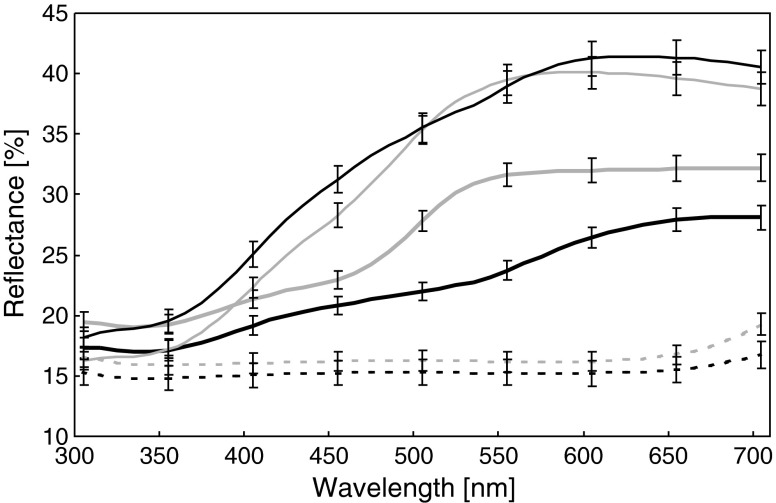
Mean (± SE) reflectance spectra of the morphs of *Cepaea nemoralis* shells. The *black thick solid line* is P0, the *black thin solid line* is the shell background of P5, the *black dotted line* is the bands of P5, the *gray thick solid line* is Y0, the *gray thin solid line* is the shell background of Y5, and the *gray dotted line* is the bands of Y5. For morph names, see the “Materials and methods” section

**Fig. 3:**
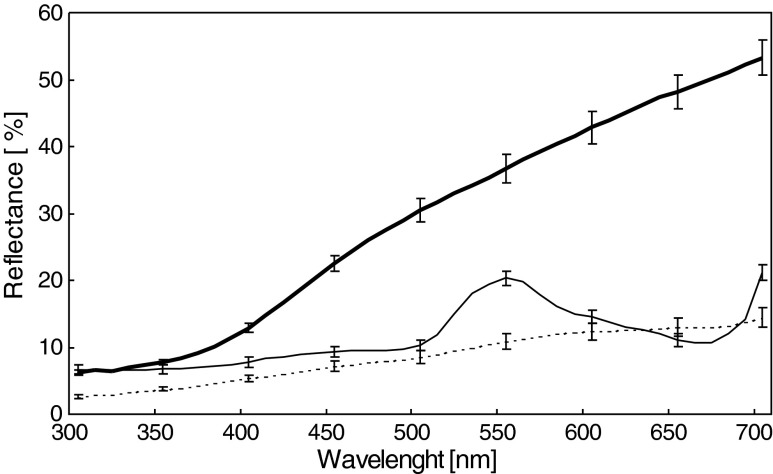
Mean (± SE) reflectance of the three habitats. The *solid thick line* is the dry vegetation, the *solid thin line* is the green vegetation, and the *dotted line* is the bare ground

To assess how thrushes Turdidae perceive snail morphs, we calculated chromatic contrast (Δ*S*) between shell and habitat colors. For banded morphs, we calculated contrasts for shell background and bands separately. The chromatic contrast (Δ*S*) is expressed in units called just noticeable differences (jnds). It is assumed that Δ*S* values >1.0 can be distinguished by birds (Vorobyev et al. 1998). Increasing values of Δ*S* suggest an increasing ability of birds to detect differences between two color patches. We calculated chromatic contrast (Δ*S*) in the following way. First, we averaged reflectance spectra from each shell region (i.e., background and bands; Fig. 2) for each individual and for each habitat background class (i.e., bare ground, dry vegatation, and green vegatation; Fig. 3). Then, we computed cone quantum catches (*Q*_i_) for each cone type using the formula provided by Vorobyev et al. (1998):

$$ {Q_i}={\smallint_{\lambda }}{R_i}\left( \lambda \right)S\left( \lambda \right)I\left( \lambda \right)O\left( \lambda \right)\mathrm{d}\lambda $$

where *λ* = a wavelength, *R*_*i*_(*λ*) = the sensitivity of cone type *i*, *S*(*λ*) = the reflectance spectrum, *I*(*λ*) = the irradiance spectrum, and *O*(*λ*) = the transmittance of the ocular media.

Members of Turdidae family, like the majority of passerines, use four cone types for color vision that are sensitive to very short (VS), short (S), medium (M), and long (L) wavelengths (Ödeen and Håstad 2003). Molecular analysis of opsins in VS cone types in the common blackbird *Turdus merula* demonstrated that they are sensitive to ultraviolet light (peak sensitivity at 369 nm; Ödeen and Håstad 2003). We used data on cone sensitivities and the transmittance of the ocular media from Hart et al. (2000) who studied blue tits *Cyanistes cearuleus*, a species with similar UV-sensitive vision. Because ambient light spectra may affect performance of avian color vision (Vorobyev et al. 1998), we computed cone quantum catches (*Q*_*i*_) using three different illuminat spectra: D65 standard daylight, the green light of forest shade, and blue sky light (Endler 1993). However, subsequent analysis of color discriminability gave very similar results for each irradiance spectrum (data not shown). Therefore, we confined our calculations to the model with D65 standard daylight.

We calculated discriminability between shell and habitat spectra using the following equation:

$$ {{{\varDelta {S^2}={{{\left( {{\omega_1}{\omega_2}} \right)}}^2}{{{\left( {\varDelta {f_4}-\varDelta {f_3}} \right)}}^2}+{{{\left( {{\omega_1}{\omega_3}} \right)}}^2}{{{\left( {\varDelta {f_4}-\varDelta {f_2}} \right)}}^2}+{{{\left( {{\omega_1}{\omega_4}} \right)}}^2}\left( {\varDelta {f_3}-\varDelta {f_2}} \right)+{{{\left( {{\omega_2}{\omega_3}} \right)}}^2}{{{\left( {\varDelta {f_4}-\varDelta {f_1}} \right)}}^2}+{{{\left( {{\omega_2}{\omega_4}} \right)}}^2}{{{\left( {\varDelta {f_3}-\varDelta {f_1}} \right)}}^2}+{{{\left( {{\omega_3}{\omega_4}} \right)}}^2}{{{\left( {\varDelta {f_2}-\varDelta {f_1}} \right)}}^2}}} \left/ {{\left( {{{{\left( {{\omega_1}{\omega_2}{\omega_3}} \right)}}^2}+{{{\left( {{\omega_1}{\omega_2}{\omega_4}} \right)}}^2}+{{{\left( {{\omega_1}{\omega_3}{\omega_4}} \right)}}^2}+{{{\left( {{\omega_2}{\omega_3}{\omega_4}} \right)}}^2}} \right)}} \right.} $$

where

$$ {{{\varDelta {f_i} = \varDelta {q_i}}} \left/ {{{q_i}}} \right.} $$

where *q*_*i*_ is the cone quantum catch (*Q*_*i*_) normalized for the irradiance spectrum and *ω*_*i*_ represents receptor noise that depends on scaling factor *T*, the relative abundance of cone types, and Weber fraction for the cone type. Scaling factor relates a proportion of the maximal cone catch to an absolute cone catch. We set *T* to 10,000 that roughly corresponds to bright illumination. We used a Weber fraction of 0.05 for all cone types and the following relative abundance of cones from blue tits: VS = 0.37, S = 0.70, M = 0.99, and L = 1.00 (Hart et al. 2000).

The Vorobyev–Osorio model assumes that color discriminability does not depend on brightness (Vorobyev et al. 1998). We therefore also calculated achromatic contrast (Δ*L*) using the formula provided by Siddiqi et al. (2004):

$$ {{{\varDelta L=\varDelta \mathrm{f_{i}}}} \left/ {\omega } \right.} $$

where

$$ \varDelta \mathrm{f_{i}}= \ln \left[ {{{{\mathrm{q_{i}}\left( {\mathrm{spec}1} \right)}} \left/ {{\mathrm{q_{i}}\left( {\mathrm{spec}2} \right)}} \right.}} \right] $$

and q_i_ indicates double cone quantum catches for two reflectance spectra (spec1 and spec2). Double cones are assumed to be involved in achromatic vision (reviewed in Cuthill 2006). We used data on double cone sensitivities of blue tits provided by Hart et al. (2000).

For each individual of Y5 and P5 morphs, we calculated weighted mean for Δ*S* and Δ*L* using methods described by Darst et al. (2006). The weight was the percentage of shell background and bands in the shell area. It was estimated by analyzing digital photographs of seven specimens of each banded morph. Because shells could be vieved by birds from various angles, we used photographs taken from above and from the side and then averaged the values. Measurements were taken using ImageJ software (http://rsbweb.nih.gov/ij/). Mean coverages (%) of shell bands and background were as follows: Y5, 45/55; P5, 42/58, respectively. Averaging contrasts for bands and shell background is justified by the way in which multicolor objects are perceived. In the case of small objects viewed from farther distance, the eye tends to average the reflectance of different parts of the object. In order to further reduce analyzed data in all morphs, we reduced Δ*S* and Δ*L* into a single variable following Darst et al. (2006). In short, the combination of chromatic Δ*S* and achromatic Δ*L* contrasts was obtained by calculating the Euclidean distance (*E*) between them, $\mathrm{E}=\sqrt{{\Delta \mathrm{S}^2+\Delta \mathrm{L}^2}}$. We performed calculations of cone quantum catches and chromatic discriminability using SPEC.01 software (Hadfield 2004).

### Statistical analysis

We verified that each variable’s distribution did not deviate significantly from normal using the Shapiro–Wilk test.

To test the effect of the microhabitat color on morph perception, we used an ANOVA with repeated measurements. The repeated measures were contrasts between the shell color of the same individual measured against three different habitats (bare ground, dry vegetation, and green vegetation). The between-subject factor was the morph type (P0, P5, Y0, and Y5). We used Tukey HSD tests for post hoc comparisons.

To assess repeatability (Lessells and Boag 1987) of spectrometer measurements, we calculated within-region repeatibility of first four measurements (Fig. 1a) done for Y0 morph (*n* = 33). Repeatabilities (*R*) of visual contrasts calculated for all background types were significant: bare soil: *R* = 0.46, *F*_1, 32_ = 4.36, *p* < 0.001; dry plant: *R* = 0.41, *F*_1, 32_ = 3.77, *p* < 0.001; green plant: *R* = 0.43, *F*_1, 32_ = 3.83, *p* < 0.001.

## Results

**Fig. 4:**
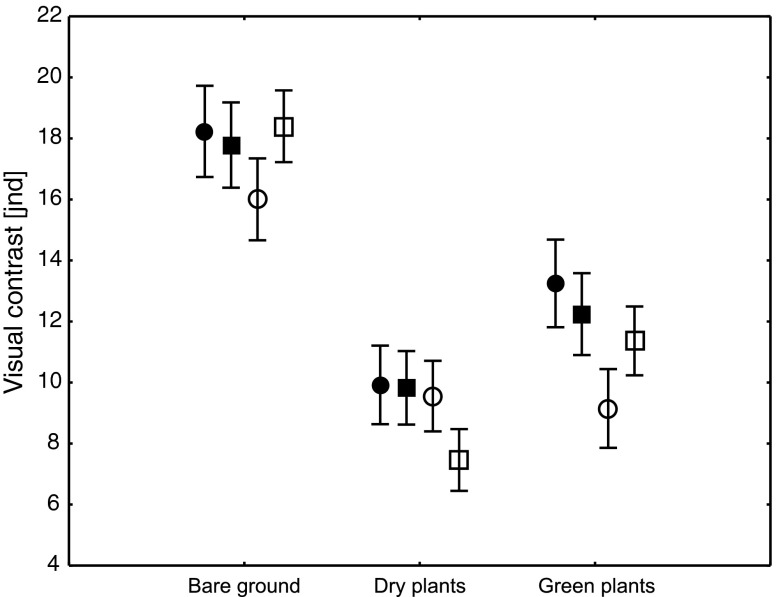
Visual contrasts measured between the shell color and the three different habitats. Morph symbols: *filled squares* Y5, *open squares* Y0, *filled circles* P5, *open circles* P0. Data are mean ± 95 % CL. For morph names, see the “Materials and methods” section

Detectability of snail shells depended significantly on morph, habitat type, and the interaction between morph and habitat (Table 1). Banded morphs tended to be more conspicuous than unbanded morphs (Fig. 4). On average, bare ground was the most conspicuous, while dry vegetation was the most cryptic microhabitat (Fig. 4). Differences in visual contrast between morphs on bare ground and dry vegetation were not statistically significant (Table 2). In the green vegatation, P0 morph was significantly more cryptic when compared to P5 and Y5 morphs (Fig. 4; Table 2). In general, P0 tended to be the most cryptic morph on bare ground and green vegatation while Y0 morph—on dry vegetation (Fig. 4). P5 was the most conspicuous morph on green vegatation (Fig. 4; Table 2).

**Table 1:** Results of a repeated-measures ANOVA comparing visual contrasts between shell and habitat colors measured for the same individual in three habitats (bare ground, dry vegetation, and green vegetation). Morph is the between-subject factor

	*F*	Df	*P*
Intercept	5,790.5	1,101	<0.01
Morph	26.6	3,101	<0.01
Habitat	274.6	2,202	<0.01
Morph × habitat	28.5	6,202	<0.01

**Table 2:** Results of Tukey’s post hoc tests comparing values of visual contrast (jnd) calculated for shell color and habitat color

	Y5—b	Y5—d	Y5—g	Y0—b	Y0—d	Y0—g	P5—b	P5—d	P5—g	P0—b	P0—d
Y5—b											
Y5—d	***										
Y5—g	***	***									
Y0—b	n.s.	***	***								
Y0—d	***	n.s.	***	***							
Y0—g	***	n.s.	n.s.	***	***						
P5—b	n.s.	***	***	n.s.	***	***					
P5—d	***	n.s	n.s.	***	n.s.	n.s.	***				
P5—g	***	*	n.s.	***	***	n.s	***	n.s.			
P0—b	n.s.	***	**	n.s.	***	***	n.s.	***	n.s.		
P0 - d	***	n.s.	n.s.	***	n.s.	n.s.	***	n.s.	*	***	
P0 - g	***	n.s.	*	***	n.s.	n.s.	***	n.s.	**	***	n.s.

Explanations: *b* bare ground, *d* dry plant, *g* green plant. For morph names, see the “Materials and methods” section

*p < 0.05; **p < 0.01; ***p < 0.001, n.s. *p* > 0.05

## Discussion

The present study is the first attempt to objectively assess crypsis of *Cepaea* land snails, the model species in surveys on animal polymorphism (Bond 2007). Based on our findings, it is not possible to explicitly categorize *Cepaea nemoralis* as an example of a specialist or as a generalist polymorphic species (*sensu* Bond 2007). In two habitats (bare ground and dry vegetation), morphs are almost equally cryptic, so predators may select morphs depending mainly on their frequency. In green vegetation, on the other hand, crypsis of morphs is markedly variable, which can be the main factor influencing predation patterns of birds. Moreover, unbanded pink and yellow morphs showed no statistically significant tendency to be more cryptic when viewed against bare ground and dry vegetation, respectively. Thus, based on the camouflage properties of *Cepaea nemoralis* shells, both apostatic selection and selection for crypsis are possible.

Selection for crypsis has been shown to be the main force maintaining color polymorphism in numerous taxa, e.g., marine snails, moths, and lizards (Hughes and Mather 1986; Ruxton et al. 2004; Stuart-Fox et al. 2004). Studies of shell crypsis in *Cepaea nemoralis* have yielded equivocal results (e.g., Cain and Sheppard 1950; 1954; Cameron 1969; Cook 1986), and our findings only partially support earlier findings. Cain and Sheppard (1950; 1954) showed that avian predation on yellow *Cepaea* is highest in the early spring and gradually decreases as the season advances and vegetation develops. This pattern was explained by changes in habitat conspicuousness for the yellow morph; conspicuousness is highest on bare ground and on dry vegetation and lowest on green vegetation (Cain and Sheppard 1950; 1954). On the other hand, Cook (1986) found no evidence that avian preference toward particular *Cepaea* morphs depended on the background color. Our study revealed that bare ground is, indeed, the most conspicuous background for the yellow morph, but in contrast, dry vegetation was the most cryptic. Moreover, all other morphs, not only the yellow unbanded morph, were most visible on bare ground. Finally, green plants provide better camouflage for pink unbanded morphs rather than for yellow morphs. These findings demonstrate how misleading conclusions can be when crypsis is estimated based on human vision.

All morphs in our study were more cryptic on vegetation compared to bare ground. High crypsis of snails on vegetation might have an adaptive value. The diet of *Cepaea nemoralis* consists mainly of dead and live plants (Richardson 1975; Williamson and Cameron 1976) on which they spend most of their time (Rosin, Z.M., personal observation). Therefore, it is advantageous for snails to be cryptic when viewed against vegetative background. On the other hand, camouflage may be less important in less favorable habitats like bare ground.

Our study does not attempt to diminish the importance of apostatic selection for *Cepaea* populations. On the contrary, the high similarity of the morphs’ crypsis in two of three studied habitats is expected for a typical “generalist” polymorphism, thought to be the result of frequency-dependent predation (Bond 2007). On the other hand, we demonstrated that relative crypsis of morphs in green vegetation differs significantly. This result underscores the importance of *Cepaea* shell crypsis in selective predation. It might be especially important when we extrapolate results from experiments of apostatic selection to wild populations. In all these experimental studies, *Cepaea* shells were exposed to predators in very simplified habitats, usually well-tended lawns (Allen and Clarke 1968; Allen 1974; Cook and Miller 1977; Tucker 1991; Allen and Weale 2005). In most of the European *Cepaea* range, common habitats are more complex both in color and structure, ranging from psammophilic habitats to hedges and deciduous woods (Cook 1998). In such a diverse background, finding prey is much more challenging for predators and may promote selection for crypsis (Cook and Kenyon 1991; Cooper and Allen 1993).

Our study provides the first quantitative measure of crypsis among *Cepaea* morphs based on color vision models of their potential avian predators. Our study supports the hypothesis that predator visual selection in addition to other important factors like, e.g., climatic selection and habitat heterogeneity (e.g., Jones 1974; Cameron and Pokryszko 2008; Ożgo 2012), is one potential cause of *Cepaea* polymorphism evolution. Findings presented in this study warrant further investigation of the role of avian predation pressure on *Cepaea nemoralis* coloration. First, visual contrast for all *Cepaea* morphs should be measured and analyzed. We may expect that the entire *Cepaea* system is even more complex than we found using four morphs. Second, we cannot exclude the possibility that mechanisms other than background matching account for shell camouflage. The dark shell bands viewed against the light shell background may create disruptive coloration and destroy the shell’s outline (Stevens and Merilaita 2009). This kind of camouflage is known to significantly reduce the likelihood that prey are detected (e.g., Merilaita and Lind 2005). Moreover, the sharply contrasting patterns of dark bands against bright (especially yellow) backgrounds may act as a conspicuous signal of the “unprofitability” of prey, similar to the Batesian mimicry of some poisonous and distasteful insects (Cott 1940; Gittleman and Harvey 1980; Endler 1981). Finally and most importantly, both experimental and correlative studies are necessary to confirm whether selection of morphs by birds follows the patterns of conspicuousness revealed by our study.
